# Small Alarmone Synthetases RelP and RelQ of *Staphylococcus aureus* Are Involved in Biofilm Formation and Maintenance Under Cell Wall Stress Conditions

**DOI:** 10.3389/fmicb.2020.575882

**Published:** 2020-09-18

**Authors:** Andrea Salzer, Daniela Keinhörster, Christina Kästle, Benjamin Kästle, Christiane Wolz

**Affiliations:** Interfaculty Institute of Microbiology and Infection Medicine, University of Tübingen, Tübingen, Germany

**Keywords:** stringent response, (p)ppGpp, biofilm, *Staphylococcus aureus*, cell wall stress

## Abstract

The stringent response is characterized by the synthesis of the alarmone (p)ppGpp. The phenotypic consequences resulting from (p)ppGpp accumulation vary among species, and for several pathogenic bacteria, it has been shown that the activation of the stringent response strongly affects biofilm formation and maintenance. In *Staphylococcus aureus*, (p)ppGpp can be synthesized by the RelA/SpoT homolog Rel upon amino acid deprivation or by the two small alarmone synthetases RelP and RelQ under cell wall stress. We found that *relP* and *relQ* increase biofilm formation under cell wall stress conditions induced by a subinhibitory vancomycin concentration. However, the effect of (p)ppGpp on biofilm formation is independent of the regulators CodY and Agr. Biofilms formed by the strain HG001 or its (p)ppGpp-defective mutants are mainly composed of extracellular DNA and proteins. Furthermore, the induction of the RelPQ-mediated stringent response contributes to biofilm-related antibiotic tolerance. The proposed (p)ppGpp-inhibiting peptide DJK-5 shows bactericidal and biofilm-inhibitory activity. However, a non-(p)ppGpp-producing strain is even more vulnerable to DJK-5. This strongly argues against the assumption that DJK-5 acts via (p)ppGpp inhibition. In summary, RelP and RelQ play a major role in biofilm formation and maintenance under cell wall stress conditions.

## Introduction

Biofilms are sessile microbial communities attached to surfaces and embedded in an extracellular matrix. Biofilm-forming staphylococci cause many device-related or chronic infections (Kong et al., 2006; O’Gara, 2007; Speziale et al., 2014; Mccarthy et al., 2015; Paharik and Horswill, 2016; Figueiredo et al., 2017; Moormeier and Bayles, 2017; Arciola et al., 2018; Otto, 2018). Depending on the composition of the biofilm matrix, staphylococcal biofilms are classified as *ica*-dependent or *ica*-independent (O’Gara, 2007). *Ica*-dependent biofilms are characterized by polysaccharide intercellular adhesin (PIA) also known as poly-N-acetyl glucosamine (PNAG), which is synthesized by enzymes encoded by the *icaADBC* operon (Götz, 2002). In *ica*-independent biofilms, proteins (Speziale et al., 2014) and extracellular nucleic acids (eDNA) (Moormeier and Bayles, 2017) are the main matrix components. Biofilm formation is a three-step process that includes initial attachment to the surface, biofilm maturation due to intercellular aggregation and bacterial cell detachment. Detachment is mediated by the enzymatic degradation of matrix components by proteases, nucleases or a group of small amphiphilic α-helical peptides, known as phenol-soluble modulins (PSMs) (Otto, 2018).

The nature and extent of biofilms are highly variable between different strains and growth conditions. Mounting evidence suggests that subinhibitory antibiotic concentrations can promote biofilm formation (Rachid et al., 2000; Kaplan et al., 2012; Liu et al., 2018; Ranieri et al., 2018; Jin et al., 2020) by inducing eDNA release and thus shifting the composition of the biofilm matrix toward a higher eDNA content (Brunskill and Bayles, 1996; Mlynek et al., 2016; Schilcher et al., 2016). Multiple regulatory mechanisms are involved in the molecular switch from a planktonic to a biofilm lifestyle, such as transcriptional regulation via SarA, SaeRS, CodY or the quorum-sensing system Agr (Kong et al., 2006; Boles and Horswill, 2008; Beenken et al., 2010; Stenz et al., 2011; Cue et al., 2012; Mrak et al., 2012; Abdelhady et al., 2014; Atwood et al., 2015; Paharik and Horswill, 2016).

For several bacterial species, it has been demonstrated that the activation of the stringent response also affects biofilm formation (Balzer and Mclean, 2002; Taylor et al., 2002; Lemos et al., 2004; Aberg et al., 2006; Chavez De Paz et al., 2012; He et al., 2012; Sugisaki et al., 2013; De La Fuente-Nunez et al., 2014; Azriel et al., 2016; Xu et al., 2016; Diaz-Salazar et al., 2017; Liu et al., 2017; Colomer-Winter et al., 2018, 2019). The stringent response is characterized by the synthesis of guanosine-tetra-phosphate (ppGpp) and guanosine-penta-phosphate (pppGpp), collectively referred to as (p)ppGpp. The accumulation of (p)ppGpp affects gene expression, protein translation, enzyme activation and replication (Wu and Xie, 2009; Liu et al., 2015; Steinchen and Bange, 2016; Bennison et al., 2019). In many pathogenic bacteria, (p)ppGpp determines virulence or antibiotic tolerance/persistence (Dalebroux et al., 2010; Hauryliuk et al., 2015; Harms et al., 2016; Wood and Song, 2020). *Staphylococcus aureus* harbors three genes encoding (p)ppGpp synthetases, Rel, RelP, and RelQ (Wolz et al., 2010). The (p)ppGpp synthetase activity of the bifunctional Rel enzyme can be induced by tRNA-synthetase inhibitors such as mupirocin or serine-hydroxamate or by amino acid deprivation (Geiger et al., 2010). Rel usually shows strong hydrolase activity, which is essential to detoxify (p)ppGpp produced by RelP or RelQ (Gratani et al., 2018). RelP and RelQ only contain a synthase domain (Geiger et al., 2014) and are part of the VraRS cell wall stress regulon (Kuroda et al., 2003). Thus, they are transcriptionally induced (e.g., after vancomycin treatment) and contribute to tolerance toward cell wall-active antibiotics such as ampicillin or vancomycin (Geiger et al., 2014).

There is some evidence that the stringent response might trigger biofilm formation in *S. aureus* based on the observation that treatment with mupirocin (Sritharadol et al., 2018; Jin et al., 2020) or serine hydroxamate (De La Fuente-Nunez et al., 2014) results in increased biofilm formation. Moreover, the anti-biofilm peptides IDR-1018 and DJK-5 have been suggested to directly interact with (p)ppGpp, preventing its signaling effects and, thus, biofilm formation (De La Fuente-Nunez et al., 2014, 2015).

Here, we aimed to investigate the role of the stringent response mediated by the two small alarmone synthetases RelP and RelQ in biofilm formation. Under cell wall stress conditions induced by a subinhibitory vancomycin concentration, both RelP and RelQ are crucial for biofilm development. Moreover, (p)ppGpp synthesis prevents biofilm eradication by vancomycin. (p)ppGpp-mediated biofilm formation was shown to be independent of the major stringent response mediator CodY and the main biofilm regulator Agr. The anti-biofilm peptide DJK-5 could prevent biofilm formation of wild type and (p)ppGpp-defective mutants. However, the (p)ppGpp^0^ strain is even more vulnerable to DJK-5. Thus, DKJ-5 does not act on biofilm formation via (p)ppGpp inhibition.

## Experimental Procedures

### Bacterial Strains and Growth Conditions

The strains and plasmids used in this study are listed in Supplementary Table S1. For overnight cultures, the strains were grown with appropriate antibiotics (10 μg/ml erythromycin, 100 μg/ml spectinomycin) at 37°C and 200 rpm. For biofilm analyses, the following media were used: chemically defined medium (CDM; Pohl et al., 2009), tryptic soy broth (TSB, Oxoid) with 3% NaCl and 0.5% glucose, and BM2 glucose [0.4% (w/v) glucose, 62 mM potassium phosphate buffer, pH 7.0, 7 mM (NH_4_)_2_SO_4_, 2 mM MgSO_4_, 10 mM FeSO_4_, 0.5% casamino acids] (De La Fuente-Nunez et al., 2014).

### Strain Construction

For *relP* complementation, the pCG833 plasmid was constructed. The complete *relP* operon with its native promoter was amplified by PCR using the primers pCG833gibfor and pCG833gibrev and cloned via Gibson assembly into the BamHI-digested integration vector pCG3. The oligonucleotides used in these procedures are listed in Supplementary Table S2. Due to showing toxicity in *E. coli*, the plasmid was directly transformed into *S. aureus* Cyl316 by electroporation. The integration of the plasmid into the genome was verified by PCR using the scv1, scv21, pCG3intfor and pCG3intrev oligonucleotides followed by sequencing. The integrated plasmid was transduced into the target strains using Φ11 phage.

For *relQ* complementation, the pCG216 plasmid was constructed. *relQ* with its native promoter was amplified by PCR with the primers BamHrelQkompl-for and BamHrelQkomp-rev and ligated with the BamHI-digested pCG3 vector backbone. The plasmid was verified by PCR with relQdigfor and relQdigrev. The plasmid was transformed into Cyl316 and verified by PCR using the primers relQfor and relQrev for integration of the plasmid. The integrated plasmid was then transduced via phage Φ11 in the target strains.

*codY* and *agr* mutations were transduced into the (p)ppGpp strain using the lysates of RN4220-21 (Pohl et al., 2009) and RN6911 (Kornblum et al., 1990), respectively.

### Colony-Forming Unit (CFU) and Minimal Inhibitory Concentration (MIC) Determination

For CFU measurements, biofilm-grown bacteria (24 h, 37°C) were resuspended by thorough pipetting. The bacterial suspension (biofilm resolved and planktonic) was serially diluted in phosphate-buffered saline (PBS), and 10 μl aliquots were spotted onto TSA plates for CFU determination. The MIC was determined by serial microdilution and *E*-tests.

### Biofilm Assay

For the static biofilm assay, 1 ml medium was inoculated in a 24-well polystyrene cell culture plate (Greiner) to obtain an OD_600_ of 0.05. After 24 h of static incubation at 37°C, the wells were washed twice with 1 ml phosphate-buffered saline (PBS, pH 7.4, Gibco). The biofilm was dried at 40°C for 30 min. For biofilm staining, 200 μl of crystal violet (80 μg/ml in distilled water) was added to the wells, followed by incubation at RT for 5 min. The wells were then washed twice with 1 ml distilled water and dried at 40°C for 30 min. Biofilm quantification was performed by A_600_ determination (microplate reader, Tecan Infinite 200 and Tecan Spark). To account for the different distributions of biofilms within a single well, measurements were performed 100 times within one well, and the average was calculated. If required, a sub-inhibitory concentration of vancomycin (0.78 μg/ml) or 5 μg/ml of the anti-biofilm peptide DJK-5 (De La Fuente-Nunez et al., 2015) were added. To test the biofilm eradication capacity, preformed biofilms (37°C, 8 h) were incubated for an additional 16 h in the presence of vancomycin at concentrations ranging from 1 to 100 μg/ml. Biofilm staining and CFU determination were performed as described above.

### Biofilm Composition

Biofilms were washed twice with PBS and treated with 1 mg/ml proteinase K (AppliChem, 37°C, 4 h), 0.1 mg/ml DNase (Sigma-Aldrich, 37°C, 4 h) or with 40 mM sodium periodate (NaIO_4_) (24 h, 4°C). The biofilms were then washed twice with PBS, dried and stained as described above.

## Results

### (p)ppGpp Synthetases Show no Significant Effect on Biofilm Formation Under Non-stress Conditions

**FIGURE 1:**
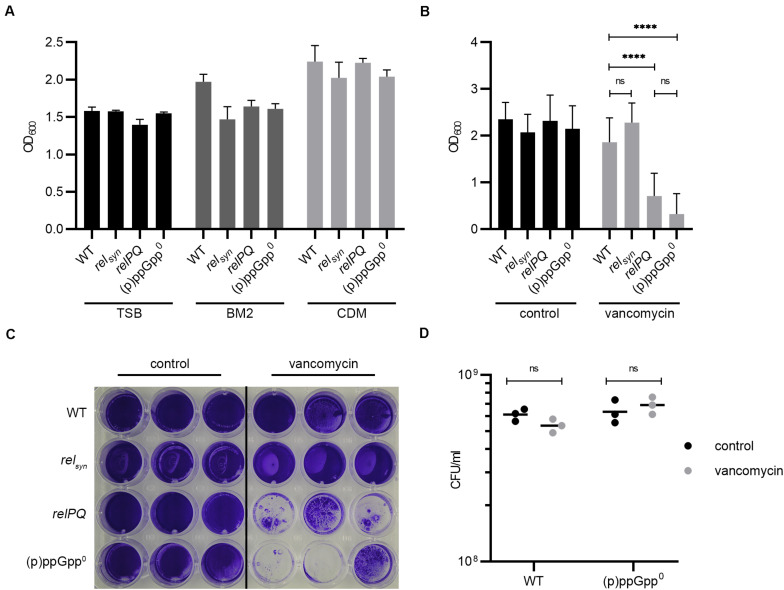
(p)ppGpp synthases effect biofilm formation under cell wall stress conditions. **(A)** Biofilm formation in TSB (+3% NaCl, +0.5% glucose), BM2 and CDM after 24 h. **(B)** Biofilm formation under uninduced and vancomycin-stress (subinhibitory vancomycin 0.78 μg/ml) conditions in CDM after 24 h. Three separate experiments were performed with biological triplicates each. Error bars represent the standard deviation, statistical significance based on ordinary one-way ANOVA (ns: not significant, ****: *P* < 0.0001). **(C)** Representative plate stained with crystal violet. **(D)** CFU was determined from resolved biofilm and planktonic bacteria after 24 h of static incubation.

Recently, it was proposed that the stringent response facilitates biofilm formation in several pathogens, including *S. aureus* (De La Fuente-Nunez et al., 2014). However, basal medium 2 (BM2) used in this study allowed hardly any *S. aureus* growth, resulting in a final OD_600_ below 1 after overnight growth. Therefore, we first analyzed the impact of (p)ppGpp on biofilm formation using different media. These media included tryptic soy broth (TSB) with the addition of 0.5% glucose and 3% NaCl, which is widely used for *S. aureus* biofilm analyses (Lade et al., 2019), and CDM (Pohl et al., 2009), used to define the stringent response phenotype in *S. aureus*. For discrimination between the Rel- and RelP/RelQ-mediated stringent response, a *rel*_*syn*_ mutant (mutation in the synthetase domain, leaving hydrolase activity unaltered), a *relP, relQ* double mutant and a (p)ppGpp^0^ mutant in which all three synthetases were non-functional were included in the analysis. Independent of the (p)ppGpp synthetases, all strains showed the strongest biofilm formation in CDM. Interestingly, in the prototypic biofilm medium TSB, biofilm formation was lower than in CDM (Figure 1A). However, no significant difference in biofilm formation was observed between the different strains in either medium. In BM2, a trend toward a slightly higher biofilm-forming capacity in the wild type compared to the mutant strains was observed, indicating that under these nutrient-limited conditions, the stringent response might be slightly activated. Thus, (p)ppGpp synthetases have no or little influence on the biofilm formation ability under non-stressed conditions.

### RelP and RelQ Regulate Biofilm Formation Under Cell Wall Stress

Subinhibitory concentrations of vancomycin were shown to transcriptionally activate *relP* and *relQ* (Geiger et al., 2014). We thus speculated that the vancomycin-induced stringent response may interfere with biofilm formation. The minimal inhibitory concentration (MIC) (1 μg/ml) of vancomycin in planktonically grown bacteria did not differ between the analyzed strains. At a subinhibitory concentration of vancomycin (0.78 μg/ml), the *relPQ* double mutant and the (p)ppGpp^0^ mutant showed significantly reduced biofilm formation compared to the wild type and the *rel*_*syn*_ mutant (Figures 1B,C). Under vancomycin treatment, the wild type formed an almost uniform thick layer of biofilm. In contrast, the *relPQ* mutant and the (p)ppGpp^0^ strain showed significantly decreased biofilm formation, with some cell aggregates remaining after the washing procedure (Figures 1B,C). The bacterial survival of the tested strains was not impaired by the subinhibitory concentrations of vancomycin (Figure 1D). Thus, the RelP- and/or RelQ-dependent changes in biofilm formation were not due to growth inhibition or bacterial killing.

### RelP and RelQ Synergistically Affect Biofilm Formation

**FIGURE 2:**
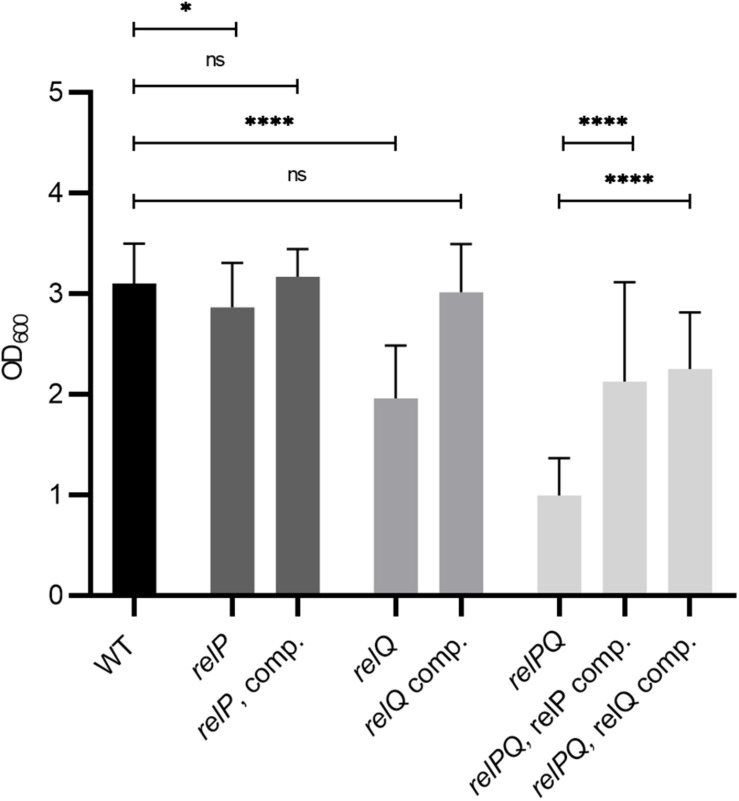
RelP and RelQ affect biofilm formation synergistically. Biofilm formation under vancomycin-stress (0.78 μg/ml vancomycin) in CDM. Strains: HG001 wild type, *relP* and *relQ* single mutants, *relPQ* double mutant and complemented strains. Three separate experiments were performed with biological triplicates each. Error bars represent the standard deviation, statistical significance based on ordinary one-way ANOVA (ns: not significant, *: *P* < 0.1, ****: *P* < 0.0001).

To determine which of the synthetases impacts biofilm formation under vancomycin conditions, single *relP* and *relQ* mutants were analyzed. Both *relP* and *relQ* contributed to biofilm formation in the presence of vancomycin. They act synergistically, since the *relPQ* double mutant showed the lowest biofilm formation (Figure 2). The *relPQ* double mutant phenotype could be complemented by the integration of either *relP* or *relQ* into the chromosome (Figure 2). Thus, the vancomycin-dependent induction of either *relP* or *relQ* is sufficient to sustain *S. aureus* biofilm formation under cell wall stress conditions.

### The Biofilm Composition Is Not Affected by the Stringent Response

**FIGURE 3:**
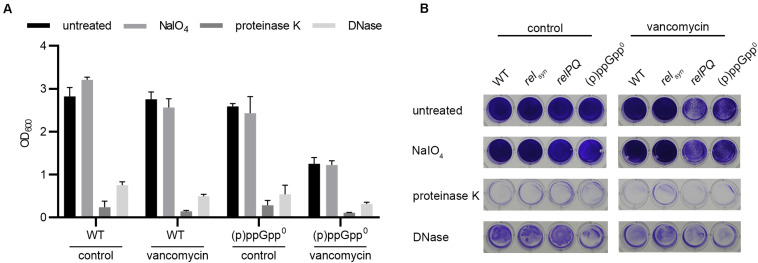
Biofilm composition is not affected by (p)ppGpp. **(A)** Preformed biofilms of the wild type and the isogenic (p)ppGpp^0^ strain were treated with either DNase or proteinase K for 4 h at 37°C or with sodium periodate for 24 h at 4°C. The remaining biofilm was stained with crystal violet and quantified by OD_600_ measurement. Three separate experiments were performed with biological triplicates each. Error bars represent the standard deviation. **(B)** Representative plate with wild type, *rel*_*syn*_, *relPQ* and the (p)ppGpp^0^ strain stained with crystal violet.

The biofilm matrix is composed of PIA, proteins or eDNA (O’Gara, 2007; Paharik and Horswill, 2016). We analyzed which matrix components were involved in the observed RelP/Q-dependent biofilm alterations under vancomycin treatment. Preformed biofilms were treated with sodium periodate, proteinase K or DNase to selectively degrade PIA, proteins or eDNA matrix components, respectively (Seidl et al., 2008). Without vancomycin, the biofilms formed by the wild type or (p)ppGpp^0^ stain were almost completely degraded by proteinase K and DNase treatment, whereas sodium periodate had no effect on the biofilm matrix (Figures 3A,B). To ensure that vancomycin does not impact the biofilm composition, we additionally examined the matrix components after vancomycin treatment. Again, the biofilms consisted almost exclusively of proteins, and eDNA and sodium periodate treatment did not degrade the biofilm matrix. Thus, under the conditions applied here, HG001 forms *ica*-independent biofilms, and the biofilm composition is not altered by the stringent response.

### The Stringent Response Induces Biofilm Formation Independent of Agr and CodY

**FIGURE 4:**
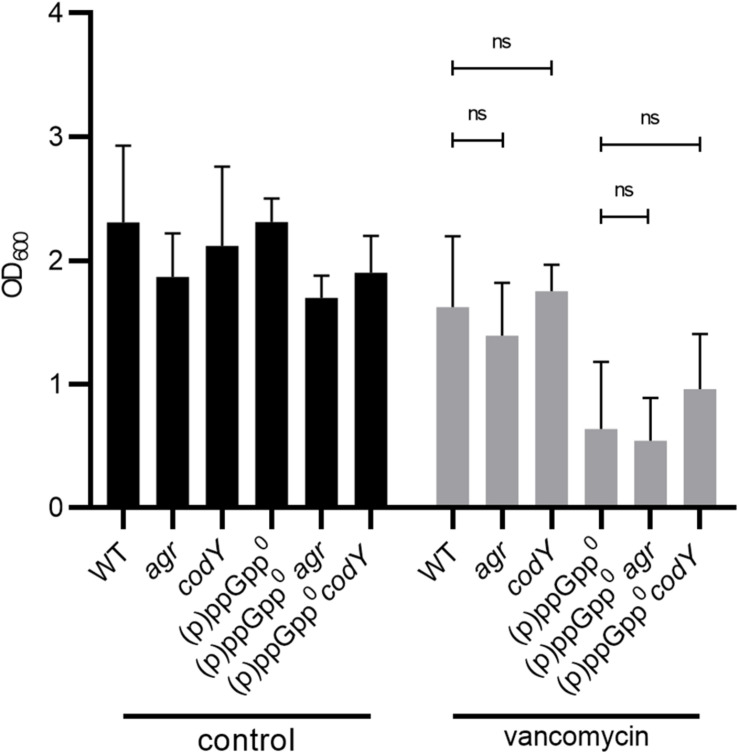
Biofilm formation under stringent conditions is independent of CodY and Agr. Biofilm formation under non-stressed and vancomycin-stress (0.78 μg/ml vancomycin) in CDM, 24 h. Three separate experiments were performed with biological triplicates each. Error bars represent the standard deviation, statistical significance based on ordinary one-way ANOVA (ns: *P* > 0.05).

The quorum-sensing system Agr (especially the target genes *psm*) (Otto, 2018) and the transcriptional regulator CodY (Stenz et al., 2011) have been identified as key controllers of biofilm structure and detachment. (p)ppGpp synthesis results in the derepression of the CodY regulon and upregulation of Agr-dependent *psm* genes (Geiger et al., 2012). Thus, we hypothesized that Agr and/or CodY activity could interfere with the observed (p)ppGpp-dependent biofilm. However, the mutation of *agr* or *codY* did not impact biofilm formation (Figure 4). Thus, under our assay conditions, biofilm formation occurs independent of CodY or Agr. Strain specific effects of Agr (Yarwood et al., 2004) or CodY (Stenz et al., 2011) on biofilm formation were described previously.

### (p)ppGpp Contributes to Biofilm Antibiotic Tolerance

**FIGURE 5:**
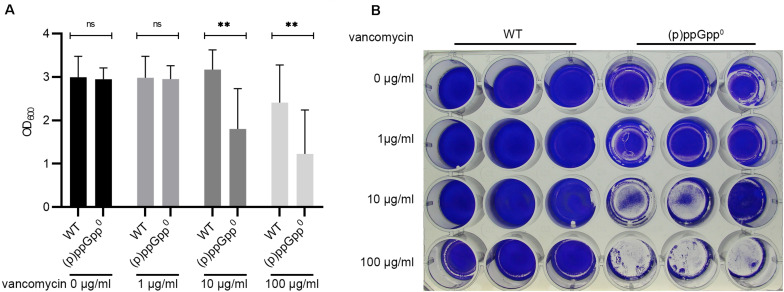
(p)ppGpp contributes to biofilm related antibiotic tolerance. **(A)** Preformed biofilms (8 h) were exposed to increasing concentrations of vancomycin for 16 h. Three separate experiments were performed with biological triplicates each. Error bars represent the standard deviation, statistical significance based on ordinary one-way ANOVA (ns: not significant, **: *P* < 0.01). **(B)** Representative plate stained with crystal violet.

Biofilms are normally more tolerant to high concentrations of antibiotics than planktonic cultures. We hypothesized that the stringent response contributes to biofilm antibiotic tolerance in *S. aureus*. Therefore, biofilm antibiotic tolerance was compared between the wild type and the isogenic (p)ppGpp^0^ mutant. Preformed biofilms were exposed to increasing concentrations of vancomycin for 16 h. At the MIC (1 μg/ml for planktonically grown bacteria), vancomycin did not result in biofilm dispersal. However, at concentrations 10- and 100-fold higher than the MIC, the biofilm produced by the (p)ppGpp^0^ strain was significantly reduced, whereas the biofilm produced by the wild type was more resistant to vancomycin treatment (Figures 5A,B). Thus, (p)ppGpp contributes to biofilm-related antibiotic tolerance.

### The Anti-Biofilm Peptide DJK-5 Exerts Its Effects Independent of (p)ppGpp

**FIGURE 6:**
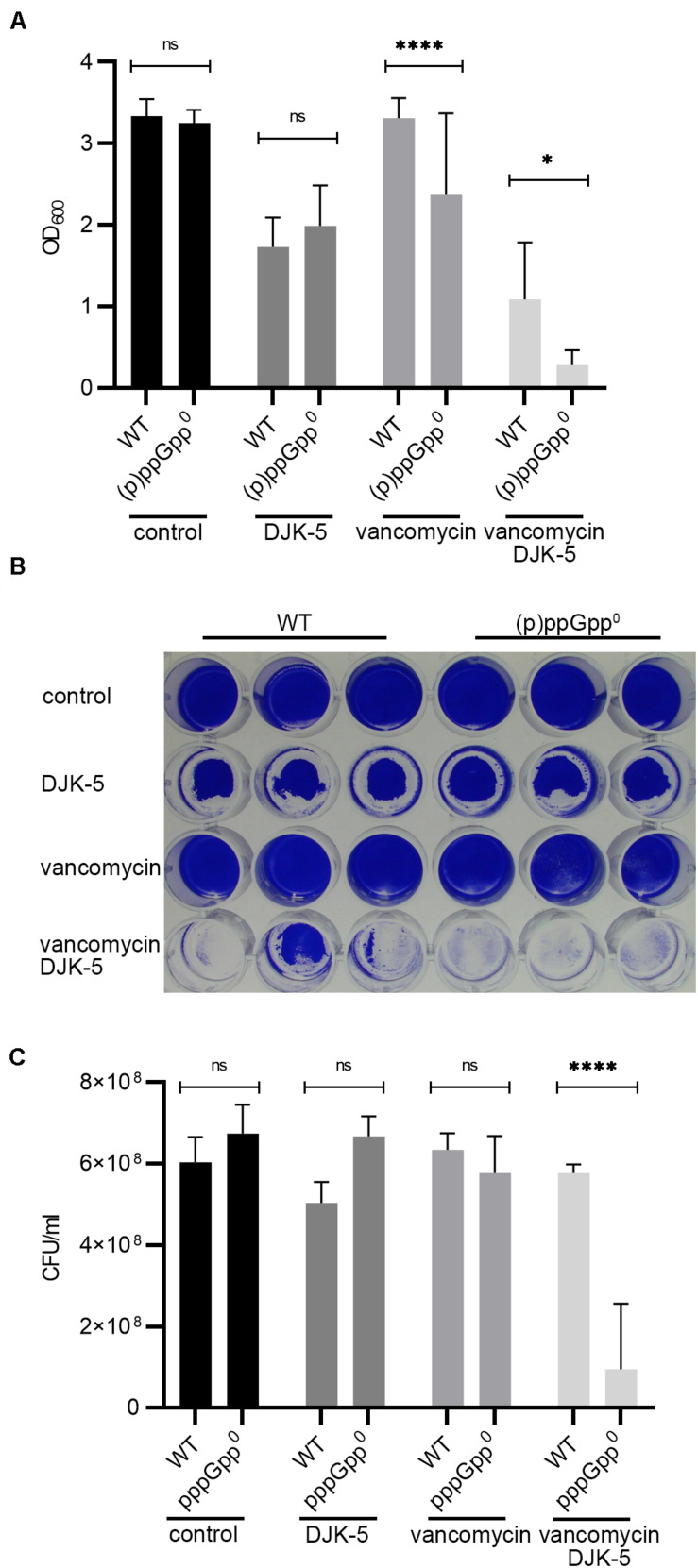
DJK-5 interferes with biofilm formation under relaxed and stringent conditions. **(A)** Biofilms grown with or without vancomycin (0.78 μg/ml) and/or the anti-biofilm peptide DJK-5 (5 μg/ml) in CDM for 24 h. **(B)** Representative plate stained with crystal violet. **(C)** CFU of planktonic and biofilm bacteria determined in parallel after static growth for 24 h. Three separate experiments were performed with biological triplicates each. Error bars represent the standard deviation, statistical significance based on ordinary one-way ANOVA (ns: not significant, *: *P* < 0.1, ****: *P* < 0.0001).

Recently, the peptides IDR-1018 (De La Fuente-Nunez et al., 2014) and DJK-5 (De La Fuente-Nunez et al., 2015) were proposed to prevent biofilms due to the specific targeting of intracellular (p)ppGpp. If correct, the peptides are expected to inhibit biofilm formation under stringent conditions in the wild type but not in the pppGpp^0^ background. We confirmed that DJK-5 interferes with biofilm formation in *S. aureus* (Figures 6A,B). However, without vancomycin treatment, biofilm formation by the wild type and pppGpp^0^ strains was equally affected by DJK-5, indicating that the effect was independent of (p)ppGpp. The combination of a subinhibitory vancomycin concentration and DJK-5 resulted in the complete inhibition of biofilm formation in the pppGpp^0^ strain. This can be explained by bacterial killing of the pppGpp^0^ strain through the synergistic action of vancomycin and DJK-5 (Figure 6C). Thus, (p)ppGpp synthesis in the wild type obviously protects the strain from the action of DJK-5. These findings are in contrast to the assumption that the biofilm-inhibiting activity of DJK-5 is exerted via (p)ppGpp inhibition, as proposed by De La Fuente-Nunez et al. (2014, 2015).

## Discussion

Here, we show that the small alarmone synthetases RelP and RelQ maintain the biofilm-forming capacity of *S. aureus* when exposed to subinhibitory concentrations of vancomycin. Both enzymes are part of the cell wall stress regulon and are transcriptionally induced by vancomycin (Geiger et al., 2014). They act synergistically, and the *relPQ* double mutant can be complemented via the chromosomal integration of either *relP* or *relQ*. Thus, the (p)ppGpp synthesis expected to occur upon vancomycin treatment supports biofilm growth, whereas without (p)ppGpp, no biofilms are formed in the presence of vancomycin. How (p)ppGpp promotes biofilm formation remains to be elucidated. (p)ppGpp results in an immediate decrease in intracellular GTP and derepression of the CodY regulon (Geiger et al., 2010). When CodY is loaded with GTP and/or branched-chain amino acids, it represses many metabolism-related genes, the Agr system and *ica* gene expression (Majerczyk et al., 2008; Pohl et al., 2009). The impact of CodY on biofilm formation is probably multifactorial and strain dependent (Stenz et al., 2011; Atwood et al., 2015). *codY* mutants have also been reported to aggregate, which can be linked with the interaction of PIA and eDNA on the bacterial surface (Mlynek et al., 2020). However, we can exclude the involvement of CodY regulation in the observed biofilm maintenance, since biofilm formation was not altered in a *codY*-negative background. Additionally, the Agr quorum-sensing system and, thus PSMs (which are strongly dependent on Agr activity), were excluded as mediators of the biofilm phenotype. Thus, the main mechanism for biofilm formation under vancomycin stress remains to be elucidated. (p)ppGpp dependent DNA-release by any of the lytic processes (e.g., autolysins, phages) likely contributes to biofilm formation. Recently, it was shown that mupirocin, a strong inducer of Rel-dependent (p)ppGpp synthesis, causes increased biofilm formation (Jin et al., 2020). Similar to our results, the mupirocin-induced biofilm forms independent of PIA and PSMs and is largely composed of eDNA. Thus, it is likely that the observed biofilm-inducing phenotype induced by mupirocin is similar to the vancomycin-dependent biofilm observed in our study. Jin et al. (2020) found that mupirocin upregulates *cidA*, encoding a holin-like protein, and that a *cidA* mutant shows reduced eDNA release. Thus, one may speculate that under our assay conditions, the (p)ppGpp-mediated activation of *cidA* may also contribute to (p)ppGpp-promoted biofilm formation.

The subinhibitory concentration of vancomycin applied in our standard biofilm assay did not affect bacterial viability, and the MIC in planktonically grown strains did not differ between the analyzed strains. When vancomycin was added to preformed biofilms, the biofilms were protected even at up to a concentration 100-fold higher than the MIC. It has been proposed that antibiotic tolerance and persister formation share common characteristics such as a slow- or non-growing phenotype (Waters et al., 2016). Here, we showed that biofilm tolerance is at least partly (p)ppGpp dependent, since biofilms of the pppGpp^0^ strain were significantly better resolved in the presence of high vancomycin concentrations. This seems to contrast with recent results indicating that (p)ppGpp is not involved in persister formation in *S. aureus* (Conlon et al., 2016). However, the persister assays were performed under relaxed conditions, and thus, the role of (p)ppGpp might have been missed. Nevertheless, (p)ppGpp synthesis was previously shown to contribute to antibiotic tolerance in *S. aureus* (Geiger et al., 2014; Bryson et al., 2020) and other pathogens (Nguyen et al., 2011; Bernier et al., 2013). Nguyen et al. (2011) suggested that in *Pseudomonas aeruginosa*, the stringent response contributes to antimicrobial tolerance in biofilms by reducing oxidative stress. We recently showed that (p)ppGpp in *S. aureus* activates ROS-detoxifying systems (Horvatek et al., 2020), which might contribute to protection against vancomycin.

Due to the role of (p)ppGpp in biofilm formation and antibiotic tolerance, the (p)ppGpp synthesis pathway is thought to be a promising antimicrobial target. Anti-biofilm peptides have been reported to exert their activity via their ability to reduce (p)ppGpp levels in live bacterial cells (De La Fuente-Nunez et al., 2014, 2015). A direct mechanism of action involving the binding of (p)ppGpp and promotion of its intracellular degradation was suggested (De La Fuente-Nunez et al., 2015). We confirmed the biofilm-dissolving effect of DJK-5. However, this was clearly not due to the proposed interaction of the peptides with (p)ppGpp because an even stronger inhibitory effect of DJK-5 was observed in the (p)ppGpp^0^ mutant. Interestingly, treatment with DJK-5 and a subinhibitory vancomycin concentration resulted in significantly higher bacterial killing activity and biofilm inhibition in the (p)ppGpp^0^ mutant than the wild type. Thus, (p)ppGpp protects against bactericidal DJK-5 activity. These findings are in good agreement with a recent re-analysis of the proposed antibiofilm peptide IDR-1018 in *E. coli* and *P. aeruginosa* (Andresen et al., 2016). Genetic disruption of the *relA* and *spoT* genes responsible for (p)ppGpp synthesis moderately sensitizes *E. coli* to IDR-1018, rather than protecting the bacterium (Andresen et al., 2016). While the IDR-1018 and DJK-5 peptides are potent antimicrobials, they do not specifically disrupt biofilms via a direct and specific interaction with the intracellular messenger nucleotide (p)ppGpp. Their alternative mode of action remains to be elucidated.

## Conclusion

In conclusion, in *S. aureus*, (p)ppGpp supports biofilm formation under cell wall stress conditions and increases tolerance against vancomycin and the anti-biofilm peptide DJK-5.

## Data Availability Statement

The original contributions presented in the study are included in the article/Supplementary Material, further inquiries can be directed to the corresponding author.

## Author Contributions

All authors listed have made a substantial, direct and intellectual contribution to the work, and approved it for publication.

## Conflict of Interest

The authors declare that the research was conducted in the absence of any commercial or financial relationships that could be construed as a potential conflict of interest.
