# Supplementary material for: Small Alarmone Synthetases RelP and RelQ of Staphylococcus aureus Are Involved in Biofilm Formation and Maintenance Under Cell Wall Stress Conditions
Source: Front Microbiol. 2020 Sep 18;11:575882. doi: 10.3389/fmicb.2020.575882 (PMC7533549; doi:10.3389/fmicb.2020.575882)
Supplement: Supplementary file 1 [file Data_Sheet_1.docx]

Supplemental Table 1: Strains and plasmids

| **Strains** | **Description** | **Reference** |
| --- | --- | --- |
| CYL316 | RN4220(pYL11219), L54 int gene, r | [1] |
| RN6911 | RN6390 *Δagr::tetM* | [2] |
| HG001 | RN1 derivative, *rsbU* repaired [3; 4] | [3] |
| HG001 (p)ppGpp^0^ | HG001 *relP/relQ/rel* mutated named as (p)ppGpp^0^ | [5] |
| HG001 *rel_syn_* | = rsh_syn_ synthetase mutated (nucleotides 942 to 950 deleted), | [6] |
| HG001 *relP relQ* | relP mutated (nucleotides 450–536 deleted) and relQ mutated (nucleotides 343–429 deleted) | [5] |
| HG001 *relP* | relP mutation (nucleotides 450–536 deleted) | [5] |
| HG001 *relQ* | relQ mutation (nucleotides 343–429 deleted) | [5] |
| HG001 *codY* | *Δ*cod Y::tet(M) | [3] |
| HG001 *agr* | *ΔagrABCD-hld* | [7] |
| HG001 (p)ppGpp^0^ *codY* | *Δ*cod Y::tet(M) | This work |
| **Plasmids** |  |  |
| pCG3 | pCL25 with erm^R^-cassette, single-copy integration vector | [8] |
| pCG833 | Integrative relP complementation plasmid | This work |
| pCG216 | Integrative relQ complementation plasmid | This work |

Supplemental Table 2 Oligonucleotides

| Primer name | sequence |
| --- | --- |
| pCG833gibfor | attcgagctcggtacccgggCATCTCTATCAATTAAGCACTTGTACCT |
| pCG833gibrev | cctgcaggtcgactctagagCAAAGTCACTCCTTCATTACACGA |
| Scv1 | GCAACACCACATAATGGTTCAC |
| Scv2.1 | TGTGCCATGATAACAGCACG |
| pCG3intfor | GCCATACCACAGATGTTCCA |
| pCG3intrev | ACATAGCGTTGCCTTGGTAG |
| relQdigfor | AAGCGGTTGATGAGTTGA |
| relQdigrev | GGCAGAAGCAGTATTT |
| BamHIrelQkompl-for | GGGGGGATCCGTTTCTGCAAAATGG |
| BamHIrelQkompl-rev | GGGGGGATCCGCATGATTTATATCG |

[1] C.Y. Lee, S.L. Buranen, and Z.H. Ye, Construction of single-copy integration vectors for *Staphylococcus aureus*. Gene 103 (1991) 101-5.

[2] R.P. Novick, S.J. Projan, J. Kornblum, H.F. Ross, G. Ji, B. Kreiswirth, F. Vandenesch, and S. Moghazeh, The *agr* P2 operon: an autocatalytic sensory transduction system in *Staphylococcus aureus*. Mol Gen Genet 248 (1995) 446-58.

[3] K. Pohl, P. Francois, L. Stenz, F. Schlink, T. Geiger, S. Herbert, C. Goerke, J. Schrenzel, and C. Wolz, CodY in *Staphylococcus aureus*: a regulatory link between metabolism and virulence gene expression. Journal of bacteriology 191 (2009) 2953-63.

[4] S. Herbert, A.K. Ziebandt, K. Ohlsen, T. Schafer, M. Hecker, D. Albrecht, R. Novick, and F. Gotz, Repair of global regulators in *Staphylococcus aureus* 8325 and comparative analysis with other clinical isolates. Infection and immunity 78 (2010) 2877-89.

[5] T. Geiger, B. Kastle, F.L. Gratani, C. Goerke, and C. Wolz, Two small (p)ppGpp synthases in Staphylococcus aureus mediate tolerance against cell envelope stress conditions. Journal of bacteriology 196 (2014) 894-902.

[6] T. Geiger, C. Goerke, M. Fritz, T. Schafer, K. Ohlsen, M. Liebeke, M. Lalk, and C. Wolz, Role of the (p)ppGpp synthase RSH, a RelA/SpoT homolog, in stringent response and virulence of *Staphylococcus aureus*. Infection and immunity 78 (2010) 1873-83.

[7] S.E. George, J. Hrubesch, I. Breuing, N. Vetter, N. Korn, K. Hennemann, L. Bleul, M. Willmann, P. Ebner, F. Gotz, and C. Wolz, Oxidative stress drives the selection of quorum sensing mutants in the Staphylococcus aureus population. Proceedings of the National Academy of Sciences of the United States of America 116 (2019) 19145-19154.

[8] M. Mainiero, C. Goerke, T. Geiger, C. Gonser, S. Herbert, and C. Wolz, Differential target gene activation by the Staphylococcus aureus two-component system saeRS. Journal of bacteriology 192 (2010) 613-23.
